# In Situ Pre-Metallization Cleaning of CoSi_2_ Contact-Hole Patterns with Optimized Etching Process

**DOI:** 10.3390/mi15121409

**Published:** 2024-11-22

**Authors:** Tae-Min Choi, Eun-Su Jung, Jin-Uk Yoo, Hwa-Rim Lee, Songhun Yoon, Sung-Gyu Pyo

**Affiliations:** School of Integrative Engineering, Chung-Ang University, 84, Heukseok-ro, Dongjak-gu, Seoul 06974, Republic of Korea; c79411@gmail.com (T.-M.C.); eunsuj@cau.ac.kr (E.-S.J.); wlsdnr5771@naver.com (J.-U.Y.); ghkfla0725@naver.com (H.-R.L.); yoonshun@cau.ac.kr (S.Y.)

**Keywords:** Ar sputtering, plasma treatment, pre-metallization cleaning, plasma induce damage, contact hole cleaning

## Abstract

We examined how controlling variables in a pre-metallization Ar sputter-etching process for in situ contact-hole cleaning affects the contact-hole profile, etching rate, and substrate damage. By adjusting process parameters, we confirmed that increasing plasma power lowered the DC bias but enhanced the etching rate of SiO_2_, while increasing RF power raised both, with RF power having a more pronounced effect. Higher Ar flow rate reduced etching uniformity and slightly lowered the DC bias. There was no significant difference in the amount of etching between the oxide film types, but the nitride/oxide selectivity ratio was about 1:2. Physical damage during Ar sputter-etching was closely linked to DC bias. finally, Finally, etching of the Si and CoSi_2_ sublayers was performed on the device contact hole model. At this time, Si losses of up to about 31.7 Å/s occurred, and the etch speed was strongly affected by the DC bias. By optimizing the RF power and plasma power, we achieved a Si/CoSi_2_ etch selectivity ratio of about 1:2.

## 1. Introduction

After the contact patterning process, etching residues often remain at the bottom of the contact holes. While most of these residues are removed through a post-etching process, the presence of a native oxide layer can persist regardless of the treatment [1,2,3]. If the oxide layer is not removed before the subsequent metallization process, it can lead to increased contact resistance and inhibit the formation of silicides with metals such as Ti and Ni [4,5], particularly if the size of the contact holes decreases. Therefore, removing the oxide layer before the contact metallization process is essential. The native oxide layer present at the bottom of the contact holes consists of a mixture of silicon oxide (SiO_2_) and sub-stoichiometric SiO_x_ (X < 2), the removal of which is known to be challenging [6,7].

Traditionally, wet-cleaning methods using hydrogen fluoride (HF)-based solutions are employed to remove the native oxide layer [8,9]. However, if the diameter of contact holes decreases (leading to an increased aspect ratio), several challenges associated with wet processing emerge. For example, line deformation can occur due to unbalanced capillary forces caused by simultaneous exposure to liquid and gas interfaces during wet processing [10,11], and the etchant may not be able to easily reach the bottom of the hole, resulting in reduced etching efficiency [12]. Moreover, it is anticipated that modifications to the overall wet-cleaning process will become necessary as device miniaturization progresses. In that context, plasma treatment such as Ar sputtering is an alternative pre-metal cleaning process [13,14,15,16,17,18,19]. It plays a crucial role in ensuring that the substrate is thoroughly cleaned before the formation of silicide, which is vital for improving the performance of Schottky barriers and overall device yield [20,21,22]. This technique not only enhances process efficiency but also allows for in situ cleaning integration [23,24].

In this study, we investigated Ar sputtering etching methods and focused on evaluating the fundamental process by observing the effects of controlling the basic process parameters for Ar sputter-etching. This involved assessing physical damage caused by the plasma charge, quantifying differences in the etching rate across various sub-materials, and examining changes in contact-hole profiles following the cleaning process.

## 2. Materials and Methods

**Figure 1:**
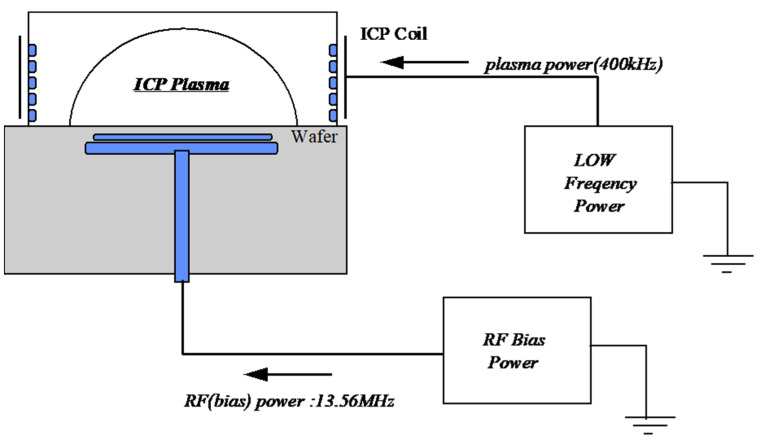
A schematic of the Ar sputter-etching chamber.

As depicted in Figure 1, the RF system in the Ar sputter-etching chamber consists of an upper inductor coil (400 kHz; coil RF supply) and an RF supply connected to the susceptor (cathode-powered supply; 13.56 MHz). The system was designed to generate a bias on the susceptor, attracting Ar ions formed by the RF coil with high bias energy. This configuration facilitates the effective removal of a native oxide layer.

First, the fundamental parameters for the Ar sputter-etching process, including RF power, plasma power, and pressure (Ar flow rate), were adjusted and the DC self-bias was measured. The etching rate and uniformity of the thermal oxide were then observed. Following this, a comprehensive evaluation was conducted, plasma damage monitoring (PDM) was used to assess the plasma charge damage on the Si surface, and physical damage was evaluated through Therma-Wave (TW) signal measurements. For this, we utilized a DOT MASK (well type: interlayer dielectric (ILD) high-temperature low-pressure dielectric (HLD) 1000 Å) lot and measurements were taken while varying the plasma power (200, 300, or 500 W) under 200 W RF power.

The samples used in the experiment had an ILD structure of nitride 500 Å/borophosphosilicate glass (BPSG) (annealed at 900 °C) 3500 Å/plasma-enhanced tetraethylorthosilicate (PETEOS) 4000 Å with a target hole size of 0.24 µm on a cobalt silicide (CoSi_2_) substrate. The samples were subjected to ion implantation processes comprising N+ (As, 30 keV, 5 × 10^15^) and P+ (BF_2_+, 30 keV, 1.5 × 10^15^). During the Ar sputter-etching process, the target etching amount was set based on 200 Å of thermal oxide, and the process time was adjusted for each experimental condition to achieve the same target etching amount.

In the case of the oxide layer, the etching characteristics of HLD, Plasma-Enhanced tetraethylorthosilicate (PETEOS), and high-density plasma (HDP) oxide were compared to BPSG. A Co-salicide protection mask was also applied to form a structure with salicide and non-salicide regions. Lastly, the contact-hole profile was observed under each set of process conditions to evaluate the degree of re-deposition and tapering changes resulting from the Ar sputter-etching process.

## 3. Results and Discussion

### 3.1. Chamber System Testing

The electromagnetic field generated by the inductively coupled plasma (ICP) coil plays a role in ionizing the Ar atoms. Consequently, as the power applied to the coil (hereafter referred to as the plasma power) is increased, the number of Ar ions in the plasma also increases [25]. During the Ar sputter-etching process, Ar ions must have energy exceeding the binding energy of oxide atoms. This enables them to move to the oxide surface and break the bonding structure [26]. To achieve this, the Ar ions ionized by the ICP RF coil must collide with the wafer surface at high velocity with sufficient energy.

After plasma is formed, ions are naturally attracted to the susceptor due to the potential difference without needing an additional potential difference to be created externally. However, in the case of Ar sputter-etching, if the process relies solely on this effect, etching may not occur due to the ion energy being lower than the threshold energy. Even if etching occurs, the etching rate of the oxide may be too low, making it difficult to achieve effective etching at the bottom of the holes in the pattern.

As shown in Figure 1, a bias on the susceptor attracts Ar ions generated by the RF coil, imparting high bias energy for effective native oxide layer removal. The etching of the native oxide can be enhanced by increasing the RF bias power applied to the susceptor, as previously described. However, as the plasma power applied to the upper coil increases, the number of ionized Ar atoms increases, even under identical DC self-bias conditions on the wafer. This effect can significantly enhance the etching rate of the native oxide layer. Therefore, one cannot consider the DC bias as the sole etching rate variable. Instead, RF power, plasma power, and the process pressure influenced by the Ar flow rate can also be determining factors for both the DC bias and the etching rate. From this perspective, Table 1 provides the trends in DC self-bias and changes in the thermal oxide etching rate when adjusting the values of these process variables. In earlier studies, the sputtering threshold ion energy for SiO_2_ has been experimentally shown to be between 25 and 40 eV [27,28,29]. Ion energy within the chamber is proportional to the combined plasma potential in the plasma region and DC self-bias [30]. In our experiments, the minimum DC self-bias of −45 V indicates that ion energy can exceed 45 eV, allowing etching to proceed smoothly beyond the threshold. The etching amount was measured based on a processing time of 30 s.

**Figure 2:**
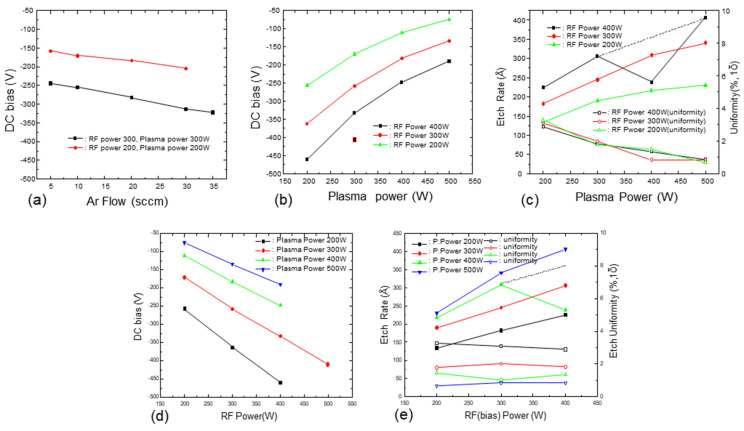
Effect of changing the etching process parameters. DC self-bias generation as a function of (**a**) Ar flow rate, (**b**) plasma power, and (**d**) RF power and etching amount as a function of (**c**) plasma power and (**e**) RF power.

Figure 2 presents the relationships between the DC bias according to RF power, plasma power, and Ar flow rate and the etching amount according to RF power and plasma power during the pre-metallization cleaning process of the thermal oxide layer. Figure 2b,c show the effects of plasma power on DC bias and etching amount, respectively, under RF power conditions of 200, 300, or 400 W. These results indicate that the DC self-bias decreased as the plasma power was increased regardless of the RF power. As previously explained, the decrease in DC self-bias occurs because more Ar atoms are ionized as the RF power applied to the upper coil is increased. This phenomenon results from the increase in RF power applied to the chamber sidewalls (as shown in Figure 1), which amplifies the RF power to the upper coil. This enhancement increases the horizontal attraction of ionized Ar toward the coil compared to the wafer surface, while reducing the vertical attraction aligned with the DC self-bias. Consequently, the Ar ions are insufficiently drawn toward the wafer and are instead directed toward the RF coil, explaining the observed reduction in DC self-bias. On the other hand, the etching rate increased as the plasma power was increased despite the decrease in DC self-bias. This can be explained by the higher the plasma power, the greater the number of Ar ions (the primary etching species). Therefore, the etching rate of the oxide layer during the pre-metallization sputter-etching process is not solely determined by DC bias but is also influenced by plasma power conditions.

The effect of RF power (bias power) on DC bias and etching rate is shown in Figure 2d and Figure 2e, respectively. Notably, increasing the RF power significantly increased both the DC self-bias and the etching rate. Similar to the interpretation of the plasma power trend, increasing the RF power resulted in a higher DC bias formed on the susceptor under the same plasma power conditions (with the same number of Ar ions) by increasing the kinetic energy of the Ar ions. It can also be observed that the RF power has a direct linear relationship with both phenomena whereas the plasma power does not. Therefore, it can be inferred that RF power has a greater impact on the Ar sputter-etching process than plasma power.

We also noted that changing the RF power had minimal impact on etching uniformity while increasing plasma power improved it. This effect is likely due to the expansion of the RF sheath area as the plasma power was increased, which is similar to increasing the plasma power and reducing the DC bias. As shown in Figure 2a, an increase in Ar flow rate (process pressure) reduces the DC bias by raising chamber pressure, which shortens the mean free path of electrons. The reduction in mean free path decreases electron collisions with the substrate, leading to a lower DC self-bias [31]. However, the increased Ar flow rate negatively impacts etching uniformity. Thus, increasing the Ar flow rate adversely affected the chamber conditions and reduced the ionization efficiency of Ar.

**Figure 3:**
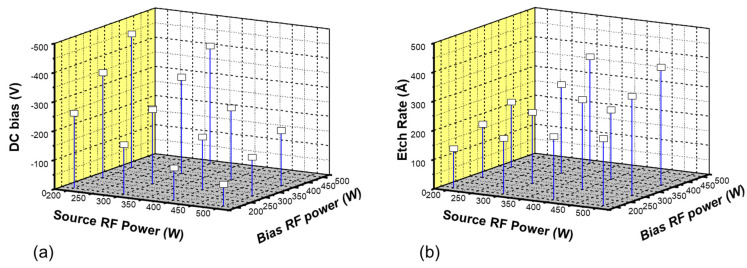
Variations in (**a**) DC bias and (**b**) sputter-etching amount as a function of RF and plasma power.

Changes in the DC self-bias and the etching rate of thermal oxide while simultaneously varying the RF and plasma power are depicted in Figure 3. As the RF power was increased and the plasma power was decreased, the DC bias on the susceptor increased. Increasing both the RF power and plasma power resulted in a higher etching amount of the oxide layer.

### 3.2. Etching of Various Oxide Layer Types

**Figure 4:**
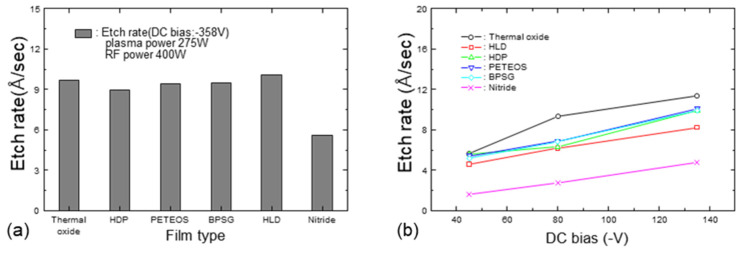
Etching rate variation with film quality. (**a**) Film type-specific etching rate under identical conditions and (**b**) DC bias-etching rate variation with film type. Tox., thermal oxide; HDP, high-density plasma; PETEOS, plasma-enhanced tetraethylorthosilicate; BPSG, borophosphosilicate glass; HLD, high-temperature low-pressure dielectric; Nit., nitride.

Figure 4a shows the etching rates for HLD, PETEOS, HDP oxide, Borophosphosilicate (BPSG), PECVD (Plasma-enhanced chemical vapor deposition) nitride films under the conditions used in the pre-metallization cleaning process employing Ar sputter-etching for current logic devices (plasma power of 275 W, RF power of 400 W, DC bias of −358 V, an Ar flow rate of 10 sccm, and a process pressure of 0.68 mtorr). The results indicate that there was no significant difference in the etching rate depending on the type of oxide film. However, a 50% reduction in the etching rate was observed for the nitride film. Although not shown in the figure, the etching uniformity for most films was approximately 2%, with negligible variation between them, except for the nitride film, which exhibited a slightly lower uniformity of 1.53% compared to the oxide films. Figure 4b presents the etching rate results for each film with the plasma power fixed at 500 W and the RF power varied as 300, 200, or 150 W, resulting in corresponding changes in DC bias of −140, −80, and −45 V, respectively. Within this ion energy range (DC bias), the etching rate decreased linearly, and the slopes were nearly identical across the different conditions. At a plasma power of 500 W, the etching rate followed the trend of thermal oxide > HDP oxide, PETEOS, BPSG > HLD. It was also noted that the nitride film exhibited a relatively low etching yield in the sputter-etching process.

### 3.3. Damage Analysis

When Ar ions (atomic mass ~40 Da) are accelerated by bias and collide with the substrate, they break the oxide bonds and remove the native oxide layer. However, at the same time, the momentum of the ions is converted into impulse energy, which can damage and cause defects in the wafer surface by etching the substrate and changing the lattice due to the implantation of Ar ions [32]. This effect can pose a particular issue not only for the ILD but also for device areas exposed during the contact process. Moreover, the non-uniform distribution of Ar ions during cleaning can result in plasma charge damage. Specifically, we investigated the effects of changes in the lattice structure along with plasma charge damage.

**Figure 5:**
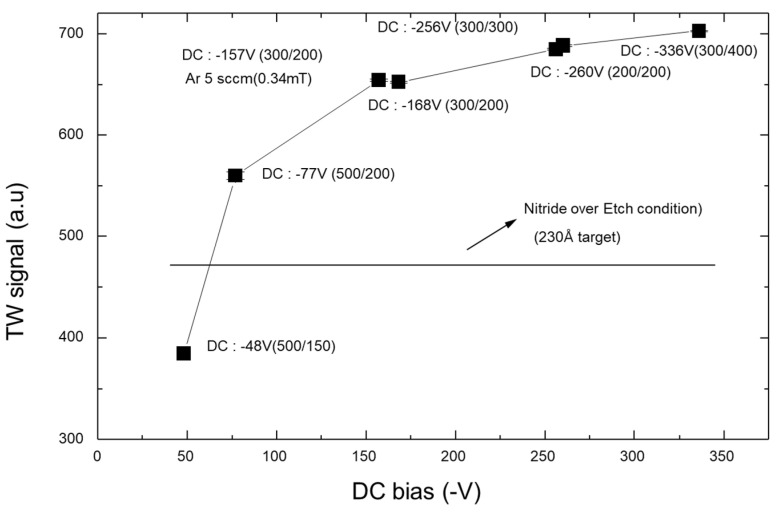
Therma-Wave (TW) signal evolution under various sets of Ar sputter-etching process conditions.

Figure 5 shows TW signals generated under various process conditions. TW is generally used to evaluate the effects of ion implantation according to the dose and applied energy by measuring changes in the thermal vibration amplitude of crystalline solids caused by impurities or lattice perturbations. In this study, the degree of crystal lattice defects caused by Ar sputter damage (where the level of damage is relatively low) was measured through the adjustment of sensitivity. Measurements were taken while varying the RF power (200, 300, or 400 W) and keeping the plasma power constant at 300 W. Lastly, the effect of process pressure was measured by setting the Ar flow rate to 5 sccm under the conditions of 300 W plasma power and 200 W RF power. Thereby, it was confirmed that the Ar sputter-etching process caused substrate damage, with the primary factor directly influencing the TW signal being the DC self-bias. For comparative observations, the TW signal was also measured under 60% nitride over-etching conditions currently applied in the contact etching process for borderless contact structures in 0.18-µm CMOS logic devices. The horizontal line in Figure 5 indicates that the TW signal value under those conditions was approximately 480; thus, the damage was greater during the sputter-etching process than during the contact opening process.

**Figure 6:**
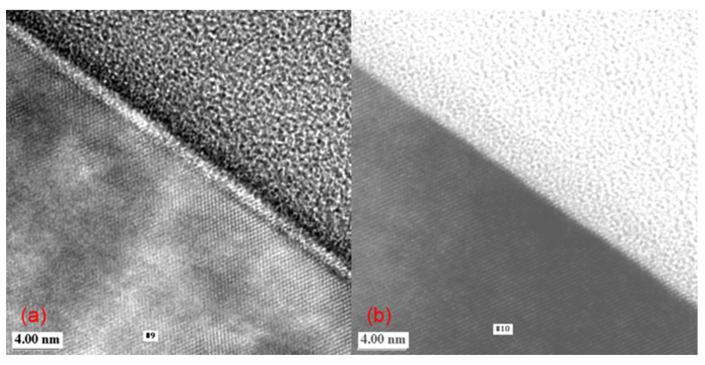
Transmission electron microscopy (TEM) images of the Si surface after (**a**) Ar sputter-etching (DC bias −77 V; RF 150 W; plasma 500 W) and (**b**) nitride over-etching (60%, 230 Å).

To better understand this phenomenon, the Si surfaces of wafers after nitride over-etching and Ar sputter-etching under a low DC bias of −77 V were examined via transmission electron microscopy (TEM) (Figure 6). It can be observed that an amorphous Si layer existed even after Ar sputter-etching with a low bias of −77 V (a TW signal of 560). This indicates that a damaged layer was formed on the Si substrate, leading to a higher bias than that during nitride over-etching (a TW signal of 480). The thickness of the damaged layer is relatively small, about 30 Å, and can be sufficiently consumed during Ti siliconization; it was not observed under the currently used process conditions, a DC bias of −358 V. Therefore, contrary to other reports, this result suggests that physical damage from Ar sputter-etching might not necessarily cause degradation of the device. However, the possibility of distortion or stress accumulation in the Si lattice beneath the amorphous layer cannot be ruled out. In addition, changes in silicidation behavior might occur, which requires verification through electrical testing.

**Figure 7:**
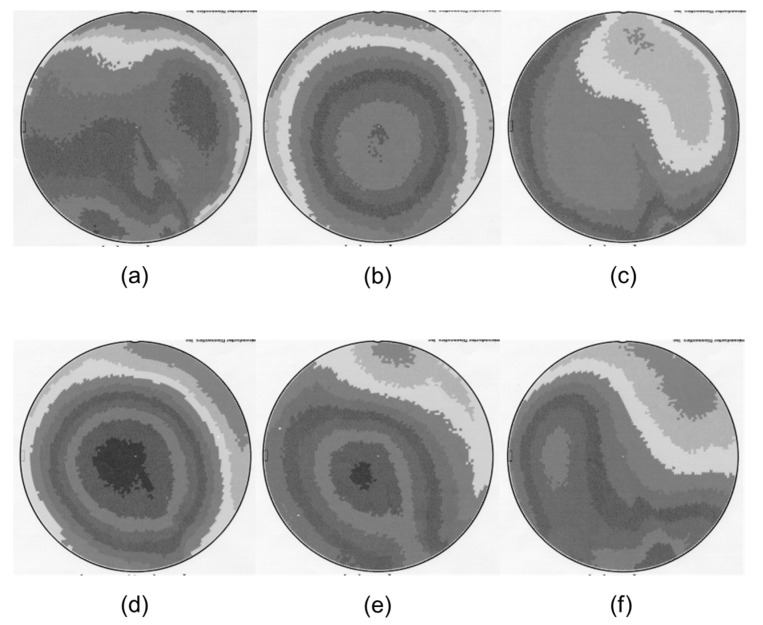
Plasma damage monitoring (PDM) results for various sets of Ar sputter-etching process conditions: (**a**) ΔV_PDM_ = 1.140 V, P 300/R 400 (DC: −333); (**b**) ΔV_PDM_ = 0.887 V, P 300/R 300 (DC −256); (**c**) ΔV_PDM_ = 0.793 V, P 200/R 200 (DC: −261); (**d**) ΔV_PDM_ = 1.458 V, P 300/R 200 (DC: −173); (**e**) ΔV_PDM_ = 0.967 V, P 500/R 200 (DC: −77); and (**f**) ΔV_PDM_ = 1.346 V, P 300/R200 (DC: −158).

Figure 7 presents the results of plasma charge damage via PDM under the same Ar sputter-etching process conditions used for the TW signal generation testing. Charging damage occurs due to the non-uniform distribution of charges when using plasma for etching or oxide deposition, specifically resulting from the difference between the ion and electron current values [33]. This phenomenon has been reported to affect gate leakage and threshold voltage. With the continued reduction in gate oxide thickness, plasma charge damage may exert a greater influence on device sensitivity. Moreover, an evaluation of the gate oxide integrity (GOI) should also be performed. From this perspective, it was observed that the change in PDM voltage (ΔV_PDM_) value remained at approximately 1 V under all sets of Ar sputter-etching conditions, indicating that significant GOI-related issues are unlikely to arise, and the current process conditions fall within a manageable range. However, none of the Ar sputter-etching process variables were found to significantly influence the PDM results, which could be due to background influences arising from device tolerance during PDM measurements or related to equipment issues that were not checked in this experiment. Therefore, a more detailed analysis of the plasma mechanism and the PDM results is required.

### 3.4. Sub-Layer Removal and Contact Profile Changes

Variations in the sub-layer etching rate and contact profile resulting from adjustments in process parameters during the Ar sputter pre-cleaning process were examined.

**Figure 8:**
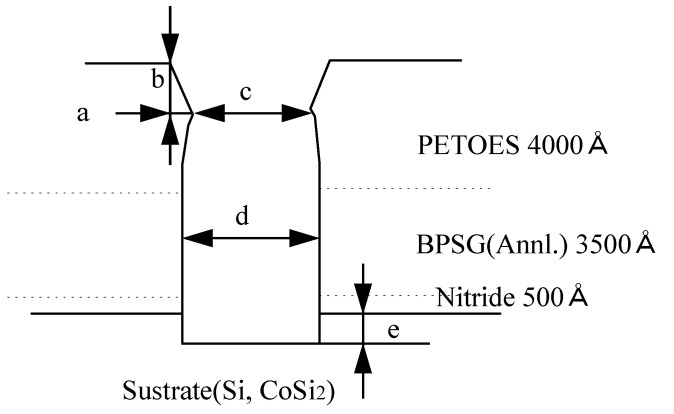
Measurement points for the Ar sputter-etching process.

As shown in Figure 8, measurement points *a* and *b* were selected to observe the degree of tapering in the contact profile after the Ar sputter-etching process and to identify the causes of bowing such as re-deposition of the oxide layer by additional etching resulting from Ar ion reflection on the sidewalls or by another factor. This ensures optimal step coverage, particularly for subsequent processes such as W or Al plugging. Measurement point *e* was used to measure the removal rate of the sub-layer. It should be noted that although the intended CoSi_2_ formation thickness was 500–600 Å, the actual thickness was only around 300 Å, which made it difficult to determine the exact removal rate.

**Figure 9:**
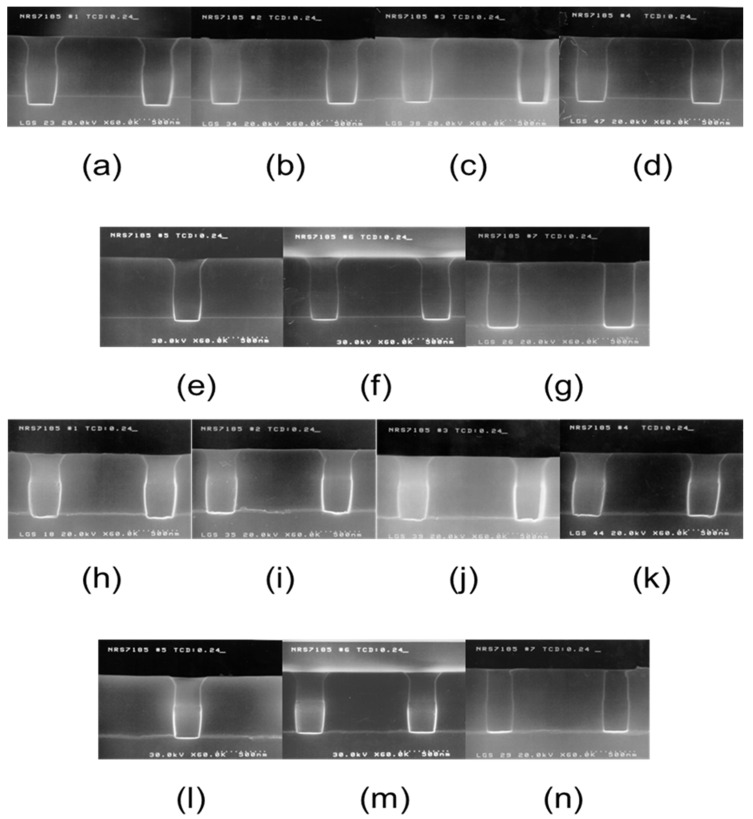
Scanning electron microscopy (SEM) images of the sub-etching amount and contact profile under various sets of Ar sputter-etching process conditions: the Si layer in (**a**) wafer #1 (P300/R400(DC-334)), (**b**) wafer #2 (P300/R300(DC-256)), (**c**) wafer #3 (P300/R200(DC-172)), (**d**) wafer #4 (P200/R200(DC-265)), (**e**) wafer #5 (P500/R200(DC-78)), (**f**) wafer #6 (P500/R150(DC-50)), and (**g**) wafer #7 (as-etched) and the CoSi_2_ layer in (**h**) wafer #1 (P300/R400(DC-334)), (**i**) wafer #2 (P300/R300(DC-256)), (**j**) wafer #3 (P300/R200(DC-172)), (**k**) wafer #4 (P200/R200(DC-265)), (**l**) wafer #5 (P500/R200(DC-78)), (**m**) wafer #6 (P500/R150(DC-50)), and (**n**) wafer #7 (as-etched).

Figure 9 shows scanning electron microscopy (SEM) images of the contact profile in a region with a CD of 0.24 µm on TP after Ar sputter-etching of a 0.18-µm CMOS logic device while Table 2 provides the values for the measurement points in Figure 8 based on the SEM images in Figure 9.

From the results in Figure 9 and Table 2, it can be deduced that there was some loss of the underlying Si layer after contact dry etching (approximately 270 Å). In addition, the contact-hole profile deviated slightly from a conformal shape, showing bowing effect. The *b*/*a* ratio values (indicating the degree of contact-hole tapering) indicate that the greatest amount of tapering was observed under plasma power of 300 W and RF power of 300 W. However, none of the process variables predominantly influenced the degree of tapering. Based on these results, we suggest that future experiments should be conducted to examine the degree of tapering while varying the process pressure. In addition, it was found that the degree of bowing in the contact-hole profile was most pronounced under the processing conditions of plasma power of 300 W and RF power of 400 W while the other sets of conditions did not cause significant bowing. The *c* and *d* measurements for wafers #1 to #6 decreased compared to the as-etched one (wafer #7). This suggests that the bowing phenomenon was likely caused by the effects of re-deposition. However, after Ar sputter-etching, the distinction between the upper PETEOS and lower BPSG layers became relatively more pronounced depending on the ILD structure. In addition, the bowing effect was most noticeable under the processing conditions for wafer #1, where the DC bias was the highest. This indicates that additional sputter effects due to the reflection of Ar ions also likely occurred. The etching extent of the sub-layer Si shows the trend: wafer #1 > wafer #3, wafer #4 > wafer #2 > wafer #5 > wafer #6. Notably, there was almost no etching loss of the sub-layer Si under the process conditions of plasma power at 500 W and RF power at 150 W, with a DC bias of 50 V. In contrast, wafer #1, under the current process conditions, exhibited the highest amount of Si loss. These results suggest that considering the increased number of Ar ions due to higher plasma power, the effect on the Si sub-layer loss largely depends on the DC bias.

Under the processing conditions for wafers #1 to #4, the CoSi_2_ layer appears to have been completely removed, with additional etching of the underlying Si layer also evident. However, under the processing conditions for wafers #5 and #6 (a lower DC bias), the CoSi_2_ layer remained. CoSi_2_ is known to have a significantly higher removal rate in Ar sputter-etching than titanium silicide (TiSi_2_). Therefore, it was necessary to perform electrical evaluations of wafers #5 and #6. Similar to the results for the Si substrate, it was confirmed that the etching behavior of CoSi_2_ also depends on the DC bias. Table 3 provides changes in the etching rate of the different sub-layers according to the process conditions to evaluate this trend quantitatively.

The results in Table 3 reaffirm that the substrate etching rate is related to the DC bias, and it was confirmed that the etching rate of CoSi_2_ was more than twice that of Si.

## 4. Conclusions

We investigated the effect of varying the parameter values of an Ar sputter-etching process to clean the contact holes in a dielectric device pre-metallization. It was discovered that plasma power and RF power significantly influenced the etching rate, contact profile behavior, and potential damage. The results demonstrate that an increase in plasma power reduced the DC bias while increasing the etching rate, whereas an increase in RF power increased both. Varying the process pressure improved etching uniformity but slightly reduced the DC bias. Notably, the selectivity between nitride and oxide was approximately 1:2, with thermal oxide exhibiting the highest etching rate. Damage evaluation indicates that greater physical damage occurred under high DC bias conditions while that caused by the plasma charge was not severe enough to cause device degradation.

In particular, we formed device contact profiles and evaluated CoSi_2_ silicide etching and sub-layer influence. Depending on the process conditions, we found Si losses of up to 31.7 Å/s Si loss at Plasma power 300 W and RF power 400 W, while only CoSi_2_ etching occurred without Si loss at Plasma power 500 W and RF power 150 W. We confirmed that Si sub-layer loss is strongly dependent on DC bias, and achieved a Si/CoSi_2_ sub-layer etching selectivity of approximately 1:2 at Plasma power 500 W and RF power 200 W condition.

Optimizing plasma power and RF power to achieve a balanced etching performance while minimizing damage presents a complex challenge in semiconductor processing. While we have provided important insights into the effects of varying the process parameters for Ar sputter-etching, several areas warrant further investigation to optimize and improve semiconductor processing. Future research should focus on understanding the combined effects of process parameter variations, hardware systems, and process optimization in Ar sputter-etching.

## Figures and Tables

**Table 1:** Etching performance for various process parameter values.

Plasma Power (W) *	RF Power (W) ^†^	Ar Flow Rate (sccm)	Pressure (mtorr)	DC Bias (V)	Etching Amount (Å)/Uniformity (%)
300	500	10	0.68	−404	366.21/1.83
200	400	10	0.68	−460	224.88/2.89
300	400	10	0.68	−332	306.29/1.82
400	400	10	0.68	−247	238.79/1.36
500	400	10	0.68	−190	406.23/0.86
200	300	10	0.68	−362	182.55/3.10
300	300	10	0.68	−258	245.31/2.01
400	300	10	0.68	−182	308.92/0.85
500	300	10	0.68	−134	340.90/0.85
200	200	10	0.68	−257	134.00/3.28
300	200	10	0.68	−170	190.19/1.78
400	200	10	0.68	−111	217.28/1.48
500	200	10	0.68	−75	230.48/0.68
500	150	10	0.68	−45	168.90/0.00
300	300	5	0.35	−244	-
300	300	10	0.68	−255	-
300	300	20	1.36	−282	-
300	300	30	2.05	−313	220.57/7.56
300	300	35	2.35	−322	-
500	300	30	2.05	−146	300.00/4.06
300	400	30	2.06	−398	272.20/6.52
300	200	5	0.31	−158	185.97/1.55
300	200	20	1.36	−183	169.79/4.32
300	200	30	2.03	−204	154.76/7.52

* RF 2nd at 400 kHz; ^†^ bias generated by the susceptor at 13.56 MHz.

**Table 2:** Changes in contact profile morphology under various sets of Ar sputter-etching process conditions.

Wafer #	*a*	*b*	*c*	*d*	*e*	CoSi_2_ Layer Etching Amount	*b/a*	*d/c*
1	800	1570	2870	3200	900 (630)	All + Si 300	1.95	1.11
2	600	1600	2970	3230	370 (100)	All	2.67	1.09
3	730	1370	2970	3230	400 (130)	All + Si 70	1.86	1.09
4	800	1330	3070	3300	400 (130)	All + Si 30	1.67	1.08
5	730	1230	2670	2930	330 (70)	200	1.68	1.10
6	800	1200	2800	3070	270 (0)	100	1.5	1.09
7	–	–	3130	3340	270	70	–	1.06

Point measurements are in Å.

**Table 3:** Comparison of sub-layer etching rates for various sets of Ar sputter-etching process conditions.

Wafer #	Plasma Power (W)	RF Power (W)	DC Bias (V)	Si Etching Rate (Å/s)	CoSi_2_ Etching Rate (Å/s)
1	300	400	−334	31.7	Etching off + Si loss
2	300	300	−256	4.0	Etching off
3	300	200	−172	4.2	Etching off + Si loss
4	200	200	−265	2.9	Etching off
5	500	200	−78	2.5	5.11
6	500	150	−50	0	0.94

## Data Availability

The original contributions presented in the study are included in the article; further inquiries can be directed to the corresponding authors.
